# Nicolau Syndrome: An Iatrogenic Cutaneous Necrosis

**DOI:** 10.4103/0974-2077.58523

**Published:** 2009

**Authors:** KC Nischal, HB Basavaraj, MR Swaroop, DP Agrawal, BD Sathyanarayana, NP Umashankar

**Affiliations:** *Department of Dermatology, Adichunchanagiri Institute of Medical Sciences, BG Nagar, Karnataka, India*

**Keywords:** Avascular necrosis, intramuscular injection, Nicolau syndrome

## Abstract

Nicolau syndrome is an uncommon complication of intramuscular injection leading to variable degrees of necrosis of skin and the underlying tissues. We report here two cases of this syndrome. Our first case was a 25 year-old male who developed intense pain and purplish discoloration of the skin in the right hip after intramuscular diclofenac injection. The second case was a 60 year-old male who developed intense pain and discoloration of skin, not only at the injection site, but also on the left scapular area and left elbow after receiving chlorpheniramine maleate injection intramuscularly. These cases highlight the need for awareness about this condition and the need to exercise utmost care during the administration of any parenteral injections by dermatologists.

## INTRODUCTION

Nicolau syndrome is an iatrogenic syndrome caused by intramuscular injection leading to variable degrees of tissue necrosis including the skin and deeper tissues.[1,2] Intense pain in the immediate postinjection period and purplish discoloration of the overlying skin, with or without a reticulate pattern, is highly characteristic of this syndrome. Intramuscular,[1‐10] subcutaneous,[11,12] intravenous,[13] and intraarticular[14] injections have been reported to produce this syndrome. The skin necrosis heals with severe and disfiguring scarring. It is therefore important that dermatologists and cutaneous surgeons are aware of this agonizing and deforming iatrogenic complication of injections.

## CASE REPORTS

### Case 1

A 25 year-old male presented with bluish discoloration of the skin of five days' duration over the right hip. The patient had generalized bodyache five days ago for which he was administered intramuscular injection of diclofenac in the right hip. He then experienced severe, dull-aching pain in the region after injection and noticed bluish discoloration of the skin. The pain regressed spontaneously and there was no history of trauma, systemic or topical medication, or spontaneous bleeding from the gingiva or mucosa.

**Figure 1:**
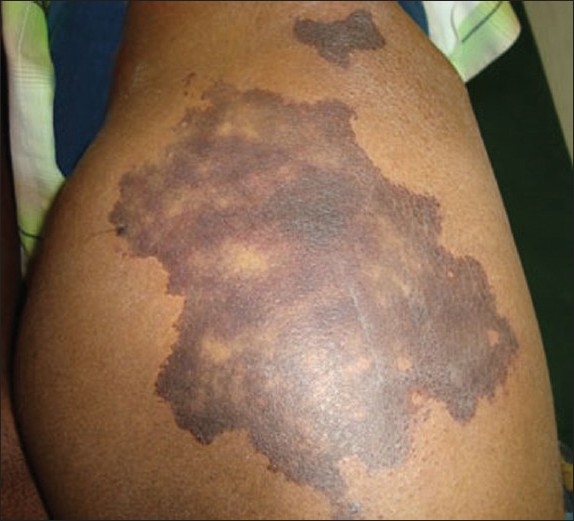
Well defined violaceous patch with reticulate pigmentation and a small satellite necrotic patch in the superomedial aspect (Case 1)

On examination, a well defined, nontender, large violaceous patch of 18 cm × 10 cm size with sharp geographic margins was found over the right hip [Figure 1]. The violaceous discoloration showed reticulate pattern and a similar smaller patch of 6 cm × 3 cm was found superomedial to the larger patch.

Based on the history and clinical features, a diagnosis of Nicolau's syndrome was made and the patient was counseled about the condition and it's course. As the manifestations were mild, it was decided to observe him before starting any aggressive treatment. He was started on systemic antibiotics to prevent secondary bacterial infection and was adviced to report after a week to check for any wound debridement. However, he was lost to follow-up.

### Case 2

A 60-year-old male presented with a nonhealing ulcer of one month's duration over the left arm. The patient had received an intramuscular chlorpheniramine maleate injection for generalized pruritus. Immediately after the injection, the patient experienced intense pain in the injection site along with bluish discoloration of the overlying skin. There was no history of any hot fomentation or application of cold compress to reduce the pain. Over the next one week, the entire skin turned black and started separating from the margins. The patient was treated for cellulitis of the arm with systemic antibiotics (Inj. Ciprofloxacin 500 mg, intravenous, BID and Inj. Metronidazole 100 mL, intravenous, TID) without much improvement. There was some history of purulent discharge and the necrotic skin denuded with the formation of the ulcer; there was no history of any systemic illnesses.

**Figure 2:**
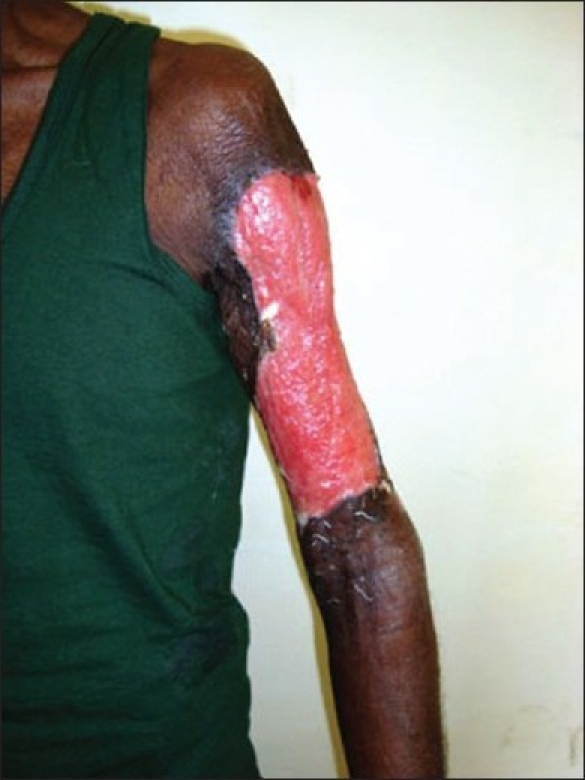
Large ulcer on the anteromedial aspect of left arm with pale unhealthy granulation tissue and slough; the margins are necrotic (Case 2)

Examination revealed a large ulcer of 15 cm × 8 cm size over the anterolateral aspect of the left arm, extending onto the posterior surface of the arm with a thick, greenish purulent discharge and pale unhealthy granulation tissue [Figure 2]. The margins were necrotic and there were blackish necrotic patches with well defined, angulated margins over the posterior deltoid region and the adjoining left back also.

**Figure 3:**
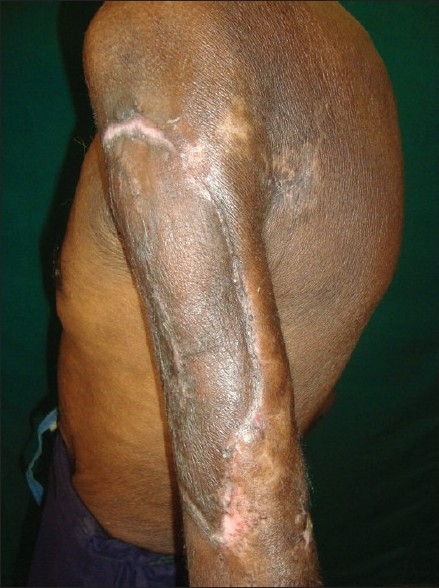
Areas where graft was not taken up has healed with wound contraction (Case 2)

**Figure 4:**
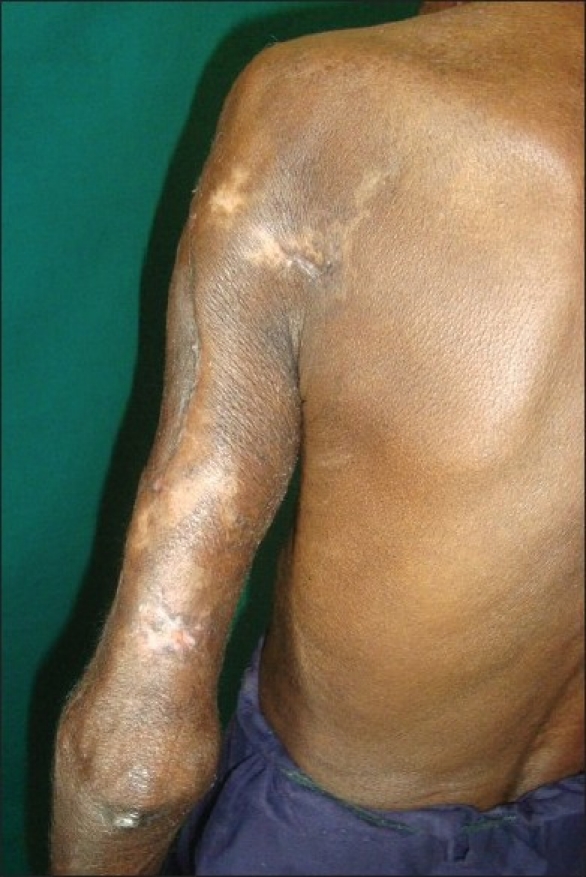
Smaller purpuric patches on the left scapular area and posterior arm have healed with hypopigmentation and scarring (Case 2)

The patient was admitted in the Dermatology ward. Investigations revealed microcytic, hypochromic anemia (hemoglobin, 8.1 g%); the results of all other serological and biochemical tests, and a roentgenogram of the chest were normal. The purulent discharge from the lesion yielded *Pseudomonas aeruginosa* on culture, which was sensitive to piperacillin. The wound was debrided and the patient started on a combination of piperacillin 4 g and tazobactam 0.5 g, intravenous BID until the culture was negative for any pathogenic growth (*i.e.,* ten days). Tab. Probenecid 500 mg BID, daily iron and vitamin supplements and a stat dose of albendazole 400 mg was administered. After daily dressings and systemic antibiotics, the wound was covered with healthy granulation in about two weeks. Split skin grafting was then done to facilitate and hasten wound healing. The graft was taken from the left anterior thigh and there was nearly 80% uptake of the graft. The rest of the ulcer healed gradually by wound contraction [Figures 3 and 4].

## DISCUSSION

Nicolau syndrome (*synonyms*: Livedo-like dermatitis, Embolia cutis medicamentosa) is an iatrogenic syndrome initially described to occur after intramuscular injections. However, there are subsequent reports of this syndrome occurring after intraarticular,[14] subcutaneous injections,[11] and intravenous injections for treating dilated veins[13] [Table 1].

**Table 1:** Various injection administration methods and medications resulting in Nicolau syndrome

Method of administration of injection	Medication injected
Intramuscular	Vitamin K,[3] NSAIDS,[4‐7] hydroxyzine,[8] vaccination,[9] bismuth,[7] benzathine penicillin,[1] penicillin G[10]
Intravenous	Polidocanol 1%[13]
Intraarticular	Glucocorticoid[14]
Subcutaneous	Pegylated interferon-α,[11] glatiramer acetate[12]
Subacromial	Triamcinolone acetate[15]

Clinically, patients with Nicolau syndrome experience intense pain at the site of injection followed by the development of bluish discoloration of the skin overlying the area of injection. The livid discoloration is well defined with sharp, angulated margins; in some, it assumes a reticulate pattern and has been referred to as noninflammatory retiform purpura,[16] livedo-like dermatitis,[7] and livedoid dermatitis with severe necrosis.[8,15] Discoloration of the skin may result in necrosis and ulceration which might involve the subcutis and the muscular layer. Paralysis of the lower extremities has been reported and attributed to embolization of the medication, mainly due to the force of injection from the gluteal vessels into the internal iliac arteries, and ischemia of sciatic nerve.[1,17] Application of cold compress tends to aggravate the tissue necrosis.[18]

The exact etiopathogenesis of the condition is poorly understood. As there is ischemic necrosis of the skin and deeper tissues, several explanations have been offered to explain the ischemia: Vasospasm secondary to needle prick, embolization of the injected material, or pressure due to the material placed around the vessel.[1] Bismuth salts, used to treat syphilis, were thought to block arterioles and cause cutaneous necrosis due to their high viscosity. However, various other less viscous agents [Table 1] have also been incriminated as causes of Nicolau syndrome.

Diagnosis is mainly clinical; skin biopsy shows necrotic changes caused by ischemia.[16,19] Ultrasonography of the skin and magnetic resonance imaging help in delineating the extent of damage. Early institution of treatment has been reported to avert necrosis of the skin.[13] In the immediate postevent period, treatment is based on various measures to improve vascularity such as pentoxyphylline, hyperbaric oxygen, intravenous alprostadil, and thrombolysis with heparin.[13] Intralesional corticosteroid has also been used to reduce inflammation.[13] Surgical debridement of the ulcer is of utmost importance as it reduces infection and enhances wound healing.

Systemic antibiotics play a vital role in the management of this syndrome. In case 2, the secondary pseudomonal infection was treated effectively with antibiotics. Probenecid was added to decrease the excretion of piperacillin and tazobactam, leading to their higher serum concentrations.[20]

Late complications include contractures and deformities resulting from the scarring process that need corrective surgery.[7] A rare complication in the form of development of soft tissue sarcoma at the site of tissue necrosis has also been reported.[21]

Aspirating just before injecting has been suggested as a method of preventing Nicolau syndrome as it is thought to help prevent embolism due to intrarterial deposition of medication. However, it is doubtful as to whether Nicolau syndrome can be prevented by this method as the spasm of the vessel or vasocompressive effect in Nicolau syndrome is usually difficult to recognize.

Nicolau syndrome has to be differentiated from necrotizing fasciitis. The latter is a rapidly spreading infection of the subcutis and fascia due to hemolytic streptococci or mixed bacteria including anaerobes. Necrotizing fasciitis occurs after surgery, or after a penetrating injury, or *de novo*. It is characterized by intense local pain with erythema of the skin which later becomes purplish or vesiculates or ulcerates. Symptoms are out of proportion to clinical signs and patients develop anesthesia of the involved skin. Often, there is presence of air in the tissues which can be detected by X-rays.

## CONCLUSION

Nicolau syndrome is an uncommon iatrogenic ischemic necrosis of the skin and deeper tissue. Although reported to occur after intramuscular injections, injections by any route can cause this syndrome. The lesions heal with scarring and deformity. As the exact etiopathogenesis of this syndrome is not known, there is no standard guideline for its management. Systemic antibiotics, wound debridement in early stages, and corrective plastic surgery in late stages are the mainstay in management.
